# Resveratrol and its synthetic derivatives exert opposite effects on mesothelial cell-dependent angiogenesis via modulating secretion of VEGF and IL-8/CXCL8

**DOI:** 10.1007/s10456-012-9266-0

**Published:** 2012-03-27

**Authors:** Justyna Mikuła-Pietrasik, Angelika Kuczmarska, Małgorzata Kucińska, Marek Murias, Marcin Wierzchowski, Marek Winckiewicz, Ryszard Staniszewski, Andrzej Bręborowicz, Krzysztof Książek

**Affiliations:** 1 Department of Pathophysiology, Poznań University of Medical Sciences, Święcickiego 6 Str., 60-781 Poznań, Poland; 2 Department of Toxicology, Poznań University of Medical Sciences, Dojazd 30 Str., 60-631 Poznań, Poland; 3 Department of Pharmaceutical Technology of Drugs, Poznań University of Medical Sciences, Grunwaldzka 6 Str., 60-780 Poznań, Poland; 4 Department of General and Vascular Surgery, Poznań University of Medical Sciences, Długa 1, 61-848 Poznań, Poland

**Keywords:** Angiogenesis, Endothelial cells, Mesothelial cells, Resveratrol, Stilbenes

## Abstract

We examined the effect of resveratrol (RVT) and its two derivatives (3,3′,4,4′-tetrahydroxy-*trans*-stilbene and 3,3′,4,4′,5,5′-hexahydroxy-*trans*-stilbene) on human peritoneal mesothelial cell (HPMC)-dependent angiogenesis in vitro. To this end, angiogenic activity of endothelial cells (HUVEC, HMVEC, and HMEC-1) was monitored upon their exposure to conditioned medium (CM) from young and senescent HPMCs treated with stilbenes or to stilbenes themselves. Results showed that proliferation and migration of endothelial cells were inhibited in response to indirect (HPMC-dependent) or direct RVT activity. This effect was associated with decreased secretion of VEGF and IL-8/CXCL8 by HPMCs treated with RVT, which confirmed the experiments with recombinant forms of these angiogenic agents. Angiogenic activity of endothelial cells treated with CM from HPMCs exposed to RVT analogues was more effective. Improved migration was particularly evident in cells exposed to CM from senescent HPMCs. Upon direct treatment, RVT derivatives stimulated proliferation (but not migration) of HUVECs, and failed to affect the behaviour of HMVEC and HMEC-1 cells. These compounds stimulated production of VEGF and IL-8/CXCL8 by HPMCs. Studies with neutralizing antibodies against angiogenic factors revealed that augmented angiogenic reactions of endothelial cells exposed to CM from HPMC treated with RVT analogues were related to enhanced production of VEGF and IL-8/CXCL8. Collectively, these findings indicate that RVT and its synthetic analogues divergently alter the secretion of the angiogenic factors by HPMCs, and thus modulate HPMC-dependent angiogenic responses in the opposite directions. This may have implications for the attempts of practical employment of the stilbenes for treatment of pathologies proceeding with abnormal vascularisation of the peritoneal tissue.

## Introduction

Peritoneal cavity is a preferential place for cancer metastases, which is especially the case for ovarian, colorectal, and pancreatic malignancies. It is believed that the reciprocal interactions between cancer cells and peritoneum-lining mesothelial cells (HPMCs) play an overriding role in this process. Aside from direct cell–cell contact mediated by certain surface molecules and their specific ligands (e.g. fibronectin-integrins β1 [1], hyaluronan-CD44 [2]), a special attention is being paid to cancer-associated angiogenesis, which—by providing appropriate supply of nutrients and removal of waste—allows tumor to overcome the critical size of 1–2 mm [3]. In fact, as evidenced in mice, primary attachment of cancer cells was restricted to highly vascularised regions of the peritoneum only [4].

Recently, a new aspect of the development of peritoneal cancer metastases, pointing on a role of senescent HPMCs, has been discovered. It has been found that senescent HPMCs—which accumulate in the peritoneum in vivo [5]—develop features that may facilitate spread of cancer. These include increased production of fibronectin and adhesion molecule ICAM-1 which mediate augmented adhesion of cancer cells [6, 7]. Moreover, senescent HPMCs secrete increased amounts of several pro-angiogenic factors whose paracrine activity corresponds to an accelerated growth of endothelial cells [8].

Resveratrol (3,4′,5-trihydroxy-*trans*-stilbene; RVT) is a naturally occurring polyphenol produced by a wide range of plants, including grapes, peanuts, and mulberries. Over the past years, this compound has attracted great deal of attention due to its anti-cancer properties. It has been found that RVT inhibits growth (by activation of p53 and/or p21 tumor suppressors) and induces apoptosis (by activation of caspases and down-regulation of anti-apoptotic proteins) in numerous malignancies, including breast, gastric, colon, prostate, and ovarian cancer (extensively reviewed in [9]). In addition, RVT has been found to increase tumor susceptibility to chemotherapeutic agents [10]. The effect of RVT on tissue vascularisation is, in turn, ambiguous and seems to depend on the specific situation [11]. In some experimental conditions, e.g. in the infracted myocardium model, RVT promoted tubular morphogenesis in coronary arteriolar endothelial cells in vitro and improved capillary density and myocardial perfusion in vivo [12, 13]. On the other hand, RVT effectively inhibited endothelial cell (HUVEC)-dependent angiogenic responses in vitro as well as reduced an excessive vascularisation in numerous malignancies in vivo [14–17]. Given an undisputable capacity of RVT to modulate tissue angiogenesis, there is an ongoing research to precisely delineate all tissue contexts in which it may reveal its stimulatory or inhibitory potential.

The activity and clinical usefulness of RVT is marred by its relatively low bioavailability and successive conversion to less active metabolites. To avoid these inconveniences, several hydroxylated and methylated RVT derivatives were synthesized, and their effectiveness in biological systems is now being intensively investigated. Significantly enough, certain properties of those analogues seem to be more pronounced compared to RVT itself [18]. At the same time, their impact on the angiogenesis remains unclear.

The study presented here was designed to examine whether RVT and its two synthetic derivatives, 3,3′,4,4′-tetrahydroxy-*trans*-stilbene and 3,3′,4,4′,5,5′-hexahydroxy-*trans*-stilbene, have an ability to modulate HPMC-dependent angiogenesis in vitro. As the ‘HPMC-dependent angiogenesis’ we understand the co-culture system in which endothelial cells proliferate and migrate in response to soluble factors released to environment (conditioned medium) by young and senescent HPMCs. Specific neutralizing antibodies and recombinant forms of angiogenic agents have been employed to identify HPMC-derived factors which may affect endothelial cell behavior, and whose secretion may be targeted by RVT and its analogues. The effect of the stilbenes on HPMC-dependent angiogenesis was confronted with a direct effect of these compounds on proliferation and migration of endothelial cells.

## Materials and methods

### Chemicals

Unless otherwise stated, all chemicals were purchased from Sigma-Aldrich Corp. (St. Louis, MO, USA). Tissue culture plastics were from Nunc (Roskilde, Denmark). Neutralizing antibodies and recombinant forms of angiogenic agents were obtained from R&D Systems (Minneapolis, MN, USA).

**Fig. 1:**
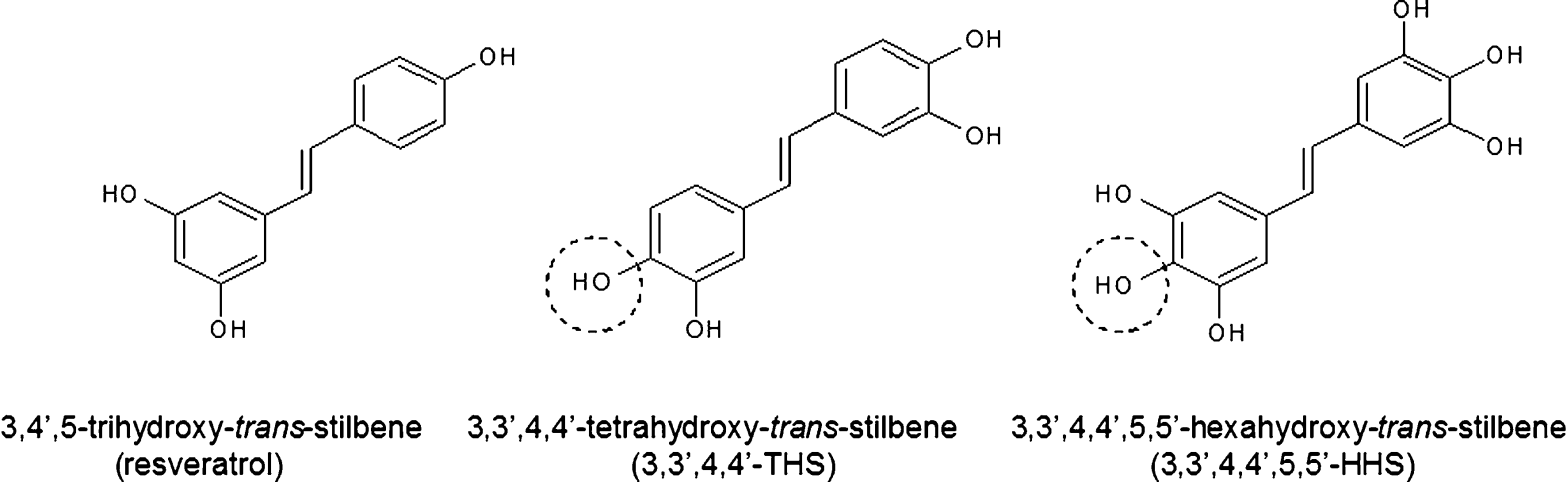
The structure of stilbenes used in the experiments. Resveratrol and its analogues differ in the number of hydroxyl (–OH) groups as well as in the presence of these groups in a highly reactive position *ortho* (marked in the *circles*)

Approximately 99 % pure resveratrol (3,4′,5-trihydroxy-*trans*-stilbene; RVT) was obtained from Sigma-Aldrich Corp. The resveratrol analogues 3,3′,4,4′-tetrahydroxy-*trans*-stilbene (3,3′,4,4′-THS) and 3,3′,4,4′,5,5′-hexahydroxy-*trans*-stilbene (3,3′,4,4′,5,5′-HHS) (Fig. 1) were synthesized in the Institute of Pharmaceutical Technology at the Poznań University of Medical Sciences, using standard chemical methodologies [19]. Stock solutions of the hydroxystilbenes were prepared in dimethyl sulfoxide (DMSO and diluted in cell culture medium to the desired final concentration. The final concentration of DMSO was always 0.05 % (v/v). During the experiments two concentrations (0.5 and 10 µM) of each stilbene were used. As shown in preliminary experiments, stilbenes used at the doses up to 10 µM did not affect HPMC viability but did markedly alter their secretory properties. Fresh culture media with stilbenes (from the stock solutions) were prepared every 3 days. Chemical stability of stilbenes in culture media was confirmed using high performance liquid chromatography (HPLC) as described in detail in [20].

### Isolation, culture and senescence of human peritoneal mesothelial cells (HPMCs)

Human peritoneal mesothelial cells (HPMCs) were isolated from the pieces of omentum, as described elsewhere [21]. Briefly, the tissue fragments were obtained from consenting patients undergoing elective abdominal surgery. The study was approved by the Ethics Committee at the Poznań University of Medical Sciences. The cells were propagated in M199 medium supplemented with l-glutamine (2 mM), penicillin (100 U/ml), streptomycin (100 μg/ml), hydrocortisone (0.4 μg/ml), and 10 % fetal bovine serum (FBS).

Upon reaching sub-confluency, cells were serially passaged (at 7-day intervals) until exhaustion of their proliferative capacity. Cultures were considered senescent when cells failed to increase in number during 4 weeks, showed enlarged morphology, and when >70 % of cells stained positively for senescence-associated β-galactosidase (SA-β-Gal) [5]. During the experiments, HPMCs were continuously exposed to resveratrol, 3,3′,4,4′-THS, and 3,3′,4,4′,5,5′-HHS, and the culture medium was exchanged every 3 days.

### Endothelial cell cultures

Human umbilical vein endothelial cells (HUVEC) were purchased in the American Type Culture Collection (Rockville, MD, USA). The cells were cultured on gelatine-coated dishes in M199 medium supplemented with 15 % FBS, l-glutamine (2 mM), HEPES (20 mM), EGF (10 μg/ml), heparin (5 U/ml), penicillin (100 U/ml), and streptomycin (100 μg/ml). Human microvascular endothelial cells (HMVEC), isolated from adult dermis, were purchased from the Clonetics Cell Systems (Lonza, Basel, Switzerland) and cultured on gelatine-coated dishes in M199 medium with 15 % FBS, l-glutamine (2 mM), EGF (10 μg/ml), heparin (5 U/ml), penicillin (100 U/ml), and streptomycin (100 μg/ml). Immortalized human dermal microvascular endothelial cells (HMEC-1) were obtained from the Center for Disease Control and Prevention (Atlanta, GA) and maintained in RPMI-1640 medium supplemented with 10 % FBS, l-glutamine (2 mM), hydrocortisone (0.4 μg/ml), penicillin (100 U/ml), and streptomycin (100 μg/ml).

In order to sensitize endothelial cells to HPMC-specific conditioned medium, HUVEC, HMVEC, and HMEC-1 cultures were exposed to M199 medium containing 10 % FBS, but deprived of supplementing growth factors, for 2 weeks before scheduled experiment. The same regimen was applied during a direct exposure of endothelial cells to the stilbenes. As observed in the preliminary studies, such a modification did not affect significantly cell viability as well as their proliferation and migration assessed in short-term conditions.

### HPMC-derived conditioned medium

Conditioned media (CM) were collected from young (first passage) and senescent HPMCs. Briefly, 3 × 10^5^ of young and senescent cells was seeded into 25 cm^5^ flasks, allowed to attach for 4 h, and incubated in serum-free medium for 72 h. The samples of CM collected were centrifuged, filtered through a 0.2 μm pore size filter to remove any cellular debris, and then stored in aliquots at −80 °C until required.

### Proliferation assay

Endothelial cells were seeded into 96-well plates at a density of 1.5 × 10^3^ cells per well, allowed to attach for 24 h, and then growth synchronized by serum deprivation for 4 h. Next, cells were exposed to HPMC-derived CM or directly to M199 medium with 10 % FBS and enriched with the stilbenes for 48-h. After that the relative cell numbers were assessed with the MTT test, as described [22]. The results obtained were normalized per number of HPMCs that gave rise to the CM at a particular passage [8]. In some experiments, endothelial cell proliferation was measured upon exposure to CM from young and senescent HPMCs enriched in exogenous recombinant forms of human VEGF, GRO-1/CXCL1, IL-8/CXCL8, and MCP-1/CCL2 as well as in specific neutralizing antibodies against those factors. The validation experiments confirmed that the results of cell proliferation obtained using the MTT assay fully correspond to those obtained according to the other methods of cell growth assessment, including a direct cell number counting, the examination of the incorporation of radiolabelled thymidine into DNA of dividing cells and the measurement of the expression of the proliferating cell nuclear antigen (PCNA).

### Migration assay

Migration assays were performed as described [23]. The lower side of 8-μm pore Transwell inserts (Costar, Inc., NY, USA) was coated with 5 μg/ml fibronectin for 1 h and blocked with 2 % bovine serum albumin in phosphate-buffered saline for 1 h. Endothelial cells (2.5 × 10^4^) were added to the upper chamber of inserts in serum-free medium. CM derived from HPMCs exposed to stilbenes or M199 medium with 10 % FBS and supplemented with the stilbenes were used as chemoattactant sources in the bottom compartment. Cells were allowed to migrate from the upper to the lower chamber for 48 h at 37  °C. Non-migratory cells were removed from the upper chamber with an absorbent tip. Cells that had migrated to the lower side of insert were fixed with 3.7 % paraformaldehyde for 15 min and with a 2 % crystal violet solution. After washing, the number of cells that had migrated were counted in three representative fields at 200× magnification. In some experiments, endothelial cell migration was measured upon exposure to CM from young and senescent HPMCs enriched in exogenous recombinant forms of human VEGF, GRO-1/CXCL1, IL-8/CXCL8, and MCP-1/CCL2 as well as in specific neutralizing antibodies against those factors. The preliminary studies concerning the robustness of the assay revealed that under the above mentioned experimental protocol (cell number, incubation time) the migration of the control (untreated) endothelial cells approximated 44 ± 8 % which corresponds to 1.1 × 10^4^ ± 2 × 10^3^ cells that migrated to the lower part of the insert. The migration effectiveness obtained for endothelial cells treated with the stilbenes has been calculated with respect to the control group which was expressed as 100 %.

### Immunoassays

Concentrations of VEGF, GRO-1/CXCL1, IL-8/CXCL8, MCP-1/CCL2 in CM from young and senescent HPMC cultures were determined using appropriate DuoSet^®^ Immunoassay Development kits (R&D Systems) according to manufacturer’s instructions.

### Inhibition studies

In order to examine the role of certain pro-angiogenic agents in regulation of HPMC-dependent endothelial cell proliferation and migration, samples of CM derived from HPMC cultures were pre-incubated at 37 °C for 2 h with specific neutralizing antibodies directed against human VEGF (5 μg/ml; AF-293-NA), GRO-1/CXCL1 (20 μg/ml; MAB275), IL-8/CXCL8 (0.5 μg/ml, MAB208), and MCP-1/CCL2 (10 μg/ml; AF-279-NA) prior application to endothelial cell cultures. In the each group of experiments, appropriate isotype-matched control mouse IgG were included. The concentrations of antibodies were chosen according to results of pilot experiments and literature data [14, 24–26].

### Measurement of reactive oxygen species (ROS)

ROS production was assessed in young and senescent HPMCs grown in 25 cm^2^ culture flasks and exposed to each stilbene for 4 h. In brief, one hundred thousand cells were incubated in the presence of 5 μM 2′,7′-dichlorodihydrofluorescein diacetate (H_2_DCFDA) (Molecular Probes, Eugene, USA) for 45 min at 37 °C. The fluorescence intensity in cell lysates was monitored in a spectrofluorimeter Victor2 (Perkin-Elmer, Turku, Finland) with excitation at 485 nm and emission at 535 nm. The results were expressed as relative light units (RLU) per 10^5^ cells.

### Statistics

Statistical analysis was performed using GraphPad Prism™ 5.00 software (GraphPad Software, San Diego, USA). The means were compared with repeated measures analysis of variance (ANOVA) with the Newman-Keuls test as post hoc. When appropriate the Wilcoxon matched pairs test was used. Results were expressed as means ± SEM. Differences with a *P* value < 0.05 were considered to be statistically significant.

## Results

### Effect of RVT and its analogues on HPMC-dependent endothelial cell proliferation

In order to evaluate the effect of stilbenes on HPMC-dependent proliferation, low density endothelial cell cultures were exposed to CM from HPMCs growing to senescence in the presence of 0.5 and 10 μM of RVT, 3,3′,4,4′-THS, and 3,3′,4,4′,5,5′-HHS, for 48 h.

**Fig. 2:**
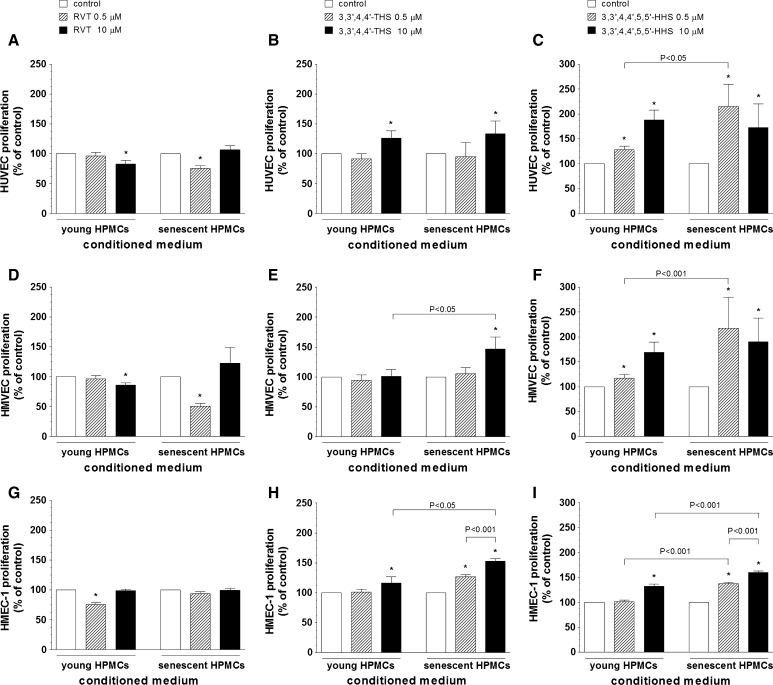
The effect of resveratrol (**A**, **D**, **G**), 3,3′,4,4′-THS (**B**, **E**, **H**) and 3,3′,4,4′,5,5′-HHS (**C**, **F**, **I**) on HPMC-dependent proliferation of endothelial cells (HUVEC, HMVEC, HMEC-1). Endothelial cells were exposed to samples of conditioned medium from young and senescent HPMCs, and their proliferation was examined using MTT test, as described in “Materials and methods”. The asterisks indicate a significant difference compared to the control group. The experiments were performed in triplicates with HPMC cultures derived from 9 to 12 different donors

As shown in the Fig. 2A, D, G, the growth capacity of endothelial cells exposed to CM from HPMCs treated with RVT was lower compared to the control group. In HUVECs and HMVECs this effect was observed upon cell exposure to CM from either young (at 0.5 μM) or senescent HPMCs (at 10 μM) while in HMEC-1 cultures it was present in response to CM from young HPMCs (at 0.5 μM) only.

Endothelial cell exposure to CM from HPMCs propagated under 3,3′,4,4′-THS yielded results shown in the Fig. 2B, E, H. This stilbene has been found to promote endothelial cell proliferation. Such a stimulatory effect was recorded for HUVECs exposed to CM from the both young and senescent HPMCs (at 10 μM), HMVECs exposed to CM from senescent HPMCs (at 10 μM), and HMEC-1 cells exposed to CM from young (at 10 μM) and senescent HPMCs (dose-dependently, at 0.5 and 10 μM). Moreover, in the case of HMVECs and HMEC-1 cells, the effects exerted by 3,3′,4,4′-THS in response to CM from senescent HPMCs were significantly greater compared with those caused by CM from young HPMCs.

The results of endothelial cell exposure to CM from HPMCs treated with 3,3′,4,4′,5,5′-HHS, depicted in the Fig. 2C, F, I, indicate that the growth promoting effects of this stilbene are even more pronounced compared to 3,3′,4,4′-THS. Proliferation of endothelial cells was elevated in response to CM from young (at 0.5 and 10 μM for HUVECs and HMVECs, and at 10 μM for HMEC-1) and senescent HPMCs (at 0.5 and 10 μM for each cell line). The effects exerted by CM from senescent HPMCs were significantly higher than those triggered by CM from young HPMCs for all endothelial cell lines studied.

### Effect of RVT and its analogues on HPMC-dependent endothelial cell migration

All three endothelial cell lines were also assayed for their migratory properties. To this end, the Transwell inserts coated with fibronectin were employed, and CM derived from HPMCs treated with the stilbenes was used as a chemoattractant source. Under these conditions, the endothelial cells were allowed to migrate for 48 h.

**Fig. 3:**
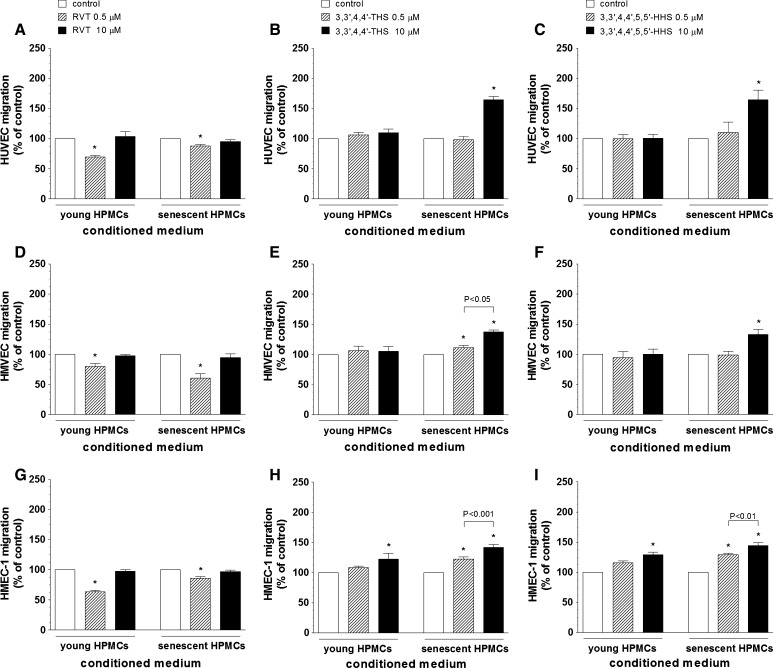
The effect of resveratrol (**A**, **D**, **G**), 3,3′,4,4′-THS (**B**, **E**, **H**) and 3,3′,4,4′,5,5′-HHS (**C**, **F**, **I**) on HPMC-dependent migration of endothelial cells (HUVEC, HMVEC, HMEC-1). Endothelial cells were exposed to samples of conditioned medium from young and senescent HPMCs, and their migration was examined using Transwell inserts, as described in “Materials and methods”. The asterisks indicate a significant difference compared to the control group. The experiments were performed in triplicates with HPMC cultures derived from 9 to 12 different donors

Results of these experiments showed that CM from HPMCs exposed to RVT markedly inhibits migration of endothelial cells. In all cell lines this effect was evident upon exposure to CM from the both young and senescent HPMCs, upon exposure to RVT at 0.5 μM (Fig. 3A, D, G).

The results of migration of endothelial cells attracted by CM from HPMCs incubated with 3,3′,4,4′-THS are shown in the Fig. 3B, E, H. The experiments revealed that this analogue exerts migration promoting activity with respect to HUVECs (CM from senescent HPMCs, at 10 μM), HMVECs (CM from senescent HPMCs, at 0.5 and 10 μM), and HMEC-1 cells (CM from young and senescent HPMCs, at 0.5 and 10 μM). In the case of HMVECs and HMEC-1 cells, a clear dose-dependency was observed in response to CM from senescent HPMC cultures.

Endothelial cell migration in response to CM from HPMCs treated with 3,3′,4,4′,5,5′-HHS is shown in the Fig. 3C, F, I. The results showed that this stilbene stimulates migration of each type of endothelial cells. In HUVECs and HMVECs cell migration was improved in response to CM from senescent HPMCs (at 10 μM) while in HMEC-1 cells the effect was present in response to CM from young (at 10 μM) and senescent HPMCs (dose-dependently, at 0.5 and 10 μM).

### Direct effect of stilbenes on proliferation and migration of endothelial cells in vitro

In order to verify of whether an indirect (HPMC-dependent) effects of stilbenes on angiogenic endothelial cell behaviour correspond to their direct activity, proliferation and migration of HUVECs, HMVECs and HMEC-1 cells were assessed in response to 48-h exposition to RVT, 3,3′,4,4′-THS and 3,3′,4,4′,5,5′-HHS. The results of these experiments are collected in the Table 1. It has been found that RVT directly applied to the cells significantly attenuates their proliferation and migration, and this effect is evident at the both concentrations used. On the other hand, 3,3′,4,4′-THS and 3,3′,4,4′,5,5′-HHS appeared to improve cell proliferation but this effect was present only in HUVECs. In the case of HMVECs and HMEC-1 cells, RVT derivatives failed to affect both aspects of their angiogenic activity.

**Table 1:** Direct effect of the stilbenes on proliferation and migration of endothelial cells

Stilbenes	Proliferation			Migration		
	HUVEC (%)	HMVEC (%)	HMEC-1 (%)	HUVEC (%)	HMVEC (%)	HMEC-1 (%)
RVT 0.5 µM	73 ± 7*	83 ± 4*	92 ± 2*	83 ± 2*	91 ± 2*	84 ± 4*
RVT 10 µM	76 ± 3*	92 ± 2*	84 ± 3*	74 ± 5*	87 ± 3*	66 ± 5*
3,3′,4,4′-THS 0.5 µM	137 ± 13*	103 ± 7	95 ± 6	105 ± 7	106 ± 6	105 ± 8
3,3′,4,4′-THS 10 µM	133 ± 15*	112 ± 11	112 ± 14	112 ± 8	116 ± 10	114 ± 14
3,3′,4,4′,5,5′-HHS 0.5 µM	128 ± 6*	106 ± 7	101 ± 6	101 ± 2	106 ± 9	114 ± 11
3,3′,4,4′,5,5′-HHS 10 µM	142 ± 6*	102 ± 5	92 ± 9	109 ± 5	98 ± 4	102 ± 9

The results are expressed as a percentage of the control (untreated) cells. The asterisks indicate a significant difference compared to the control group. The results derive from 8 to 12 experiments performed for each type of endothelial cells in triplicates

### Effect of RVT on secretion of angiogenic agents by young and senescent HPMCs

**Fig. 4:**
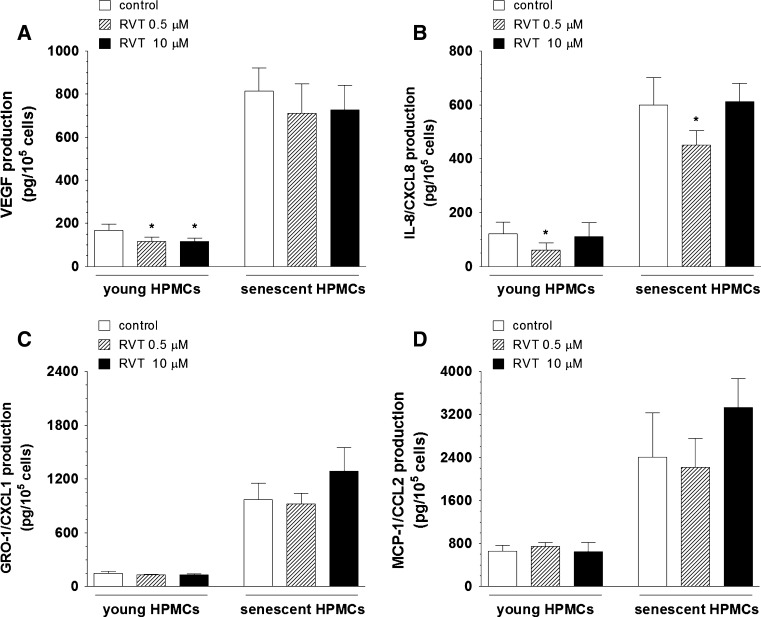
The effect of resveratrol (RVT) on secretion of VEGF (**A**), IL-8/CXCL8 (**B**), GRO-1/CXCL1 (**C**), and MCP-1/CCL2 (**D**) by young and senescent HPMCs. The asterisks indicate a significant difference compared to the control group. The experiments were performed in triplicates with HPMC cultures derived from 9 different donors

HPMCs are known to constitutively secrete wide-array of angiogenic factors, including VEGF, IL-8/CXCL8, GRO-1/CXCL1, and MCP-1/CCL2. Therefore, in order to examine of whether an inhibited proliferation of endothelial cell exposed to CM from HPMCs treated with RVT may be related to altered production of certain angiogenic mediators, the specific ELISA kits were employed to assess the angiogenic agent release into the environment (CM) by young and senescent HPMCs. The measurements revealed that the production of VEGF by young HPMCs and the production of IL-8/CXCL8 by young and senescent HPMCs exposed to RVT were markedly decreased. The effect on VEGF was found at 0.5 and 10 μM of RVT while reduction in IL-8/CXCL8 was observed at the concentration of 0.5 μM (Fig. 4A, B). At the same time, the measurements of GRO-1/CXCL1 and MCP-1/CCL2 secretion by HPMCs in response to RVT showed that their release remained unchanged either in young or in senescent cultures, irrespectively on the stilbene concentration used (Fig. 4C, D).

### Effect of exogenous recombinant forms of angiogenic agents on HPMC-dependent endothelial cell proliferation and migration

**Fig. 5:**
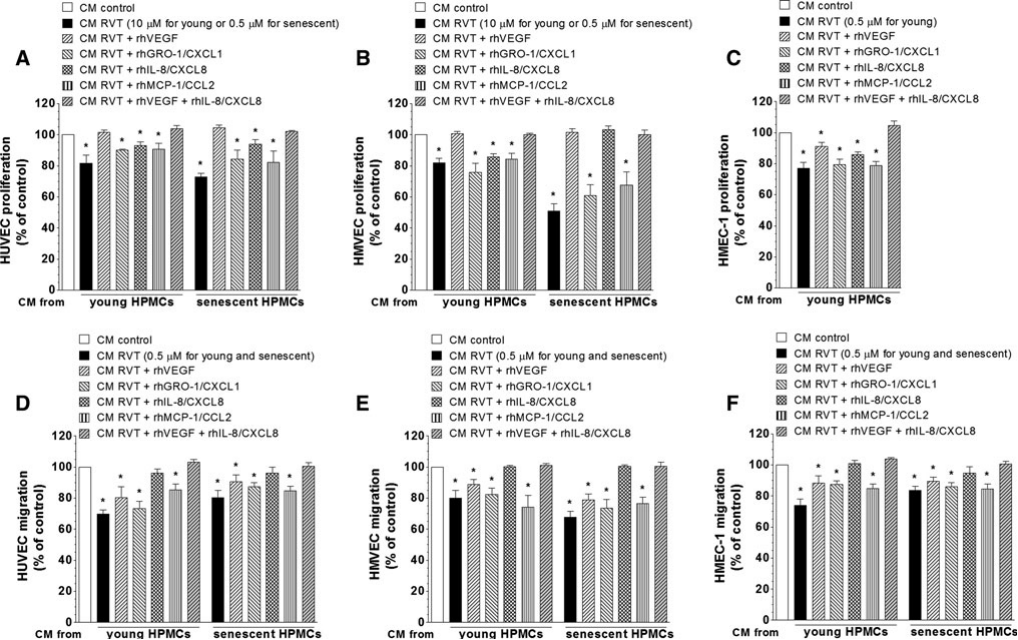
The effect of exogenous recombinant forms of angiogenic agents on proliferation and migration of endothelial cells in response to conditioned medium (CM) from HPMCs treated with resveratrol (RVT). Endothelial cell cultures were exposed to CM from HPMCs treated with RVT, and supplemented with recombinant human VEGF, GRO-1/CXCL1, IL-8/CXCL8, MCP-1/CCL2. Afterwards their proliferation (**A**–**C**) and migration (**D**–**F**) were assessed as described in “Materials and methods”. In the case of HMEC-1 cells, their proliferation was examined only with respect to CM from young HPMCs since the exposure to CM from senescent cultures treated with RVT did not affect cell growth (see Fig. 2G). The asterisks indicate a significant difference compared to the control group. The experiments were performed in triplicates with HPMC cultures derived from 9 to 12 different donors

In order to additionally confirm an involvement of certain pro-angiogenic agents in RVT-dependent inhibition of endothelial cell proliferation and migration, HUVECs, HMVECs and HMEC-1 cells were exposed to CM from HPMCs supplemented with exogenous recombinant forms of human VEGF, IL-8/CXCL8, GRO-1/CXCL1, and MCP-1/CCL2. Each angiogenic agent was applied at the dose corresponding to the difference between its concentration in control CM and in CM from HPMCs exposed to RVT. Results depicted in the Fig. 5 showed that regarding proliferation, growth capacity of HUVECs and HMVECs can be restored to the values characterizing the control group by exogenous VEGF or by its combination with exogenous IL-8/CXCL8. In the case of HMEVC cultures exposed to CM from senescent HPMCs, similar effect can also be reached by an addition of exogenous IL-8/CXCL8. With respect to HMEC-1 cells, in turn, their proliferation can be restored by the combination of exogenous VEGF and IL-8/CXCL8 only (panels A, B, C).

At the same time, in each type of endothelial cells their migratory potential can be equated with the control values only in response to exogenous IL-8/CXCL-8 or, alternatively, by combination of this chemokine with recombinant VEGF (panels D, E, F).

### Effect of RVT analogues on secretion of angiogenic agents by young and senescent HPMCs

**Fig. 6:**
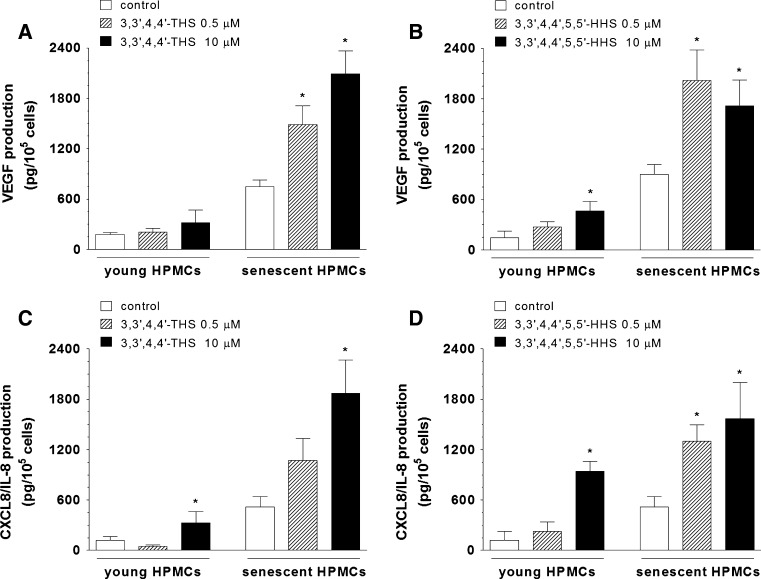
The effect of 3,3′,4,4′-THS (**A**, **C**) and 3,3′,4,4′,5,5′-HHS (**B**, **D**) on the production of VEGF and IL-8/CXCL8 by young and senescent HPMCs. The asterisks indicate a significant difference compared to the control group. The experiments were performed in triplicates with HPMC cultures derived from 8 different donors

We set out to examine the effect of 3,3′,4,4′-THS and 3,3′,4,4′,5,5′-HHS on production of angiogenesis mediators by young and senescent HPMCs. Results showed that 3,3′,4,4′-THS dose-dependently up-regulates secretion of VEGF by senescent HPMCs and stimulates production of IL-8/CXCL8 by young and senescent HPMCs, when used at the concentration of 10 μM (Fig. 6A, C). HPMC exposure to 3,3′,4,4′,5,5′-HHS resulted in an elevated release of VEGF and IL-8/CXCL8 (in young cells at 10 μM and in senescent cells at 0.5 and 10 μM) (Fig. 6B, D). At the same time there was no effect of both stilbenes on production of GRO-1/CXCL1 and MCP-1/CCL2 by either young or senescent HPMCs, irrespectively on the stilbene concentration used (data not shown).

### Effect of neutralizing antibodies against angiogenic factors on HPMC-dependent proliferation and migration of endothelial cells

Studies with specific neutralizing antibodies directed against VEGF, GRO-1/CXCL1, IL-8/CXCL8, MCP-1/CCL2 were employed to identify HPMC-derived pro-angiogenic factors which may be responsible for increased proliferation and migration of endothelial cells in response to CM from HPMCs treated with 3,3′,4,4′-THS and 3,3′,4,4′,5,5′-HHS.

As shown in the Table 2, the proliferative capacity of HUVECs and HMEC-1 cells was effectively reduced when the samples of CM derived from HPMCs exposed to 3,3′,4,4′-THS or 3,3′,4,4′,5,5′-HHS were pre-incubated with the neutralizing antibodies against VEGF and the chemokine IL-8/CXCL8. In the experiments on HMVEC cultures, their proliferation was significantly inhibited only in response to anti-VEGF antibodies added to CM from young and senescent HPMCs. At the same time, the neutralization of GRO-1/CXCL1 and MCP-1/CCL2 failed to affect cell proliferation in response to both RVT derivatives, irrespectively on endothelial cell type used and replicative age of HPMCs from whom CM samples were obtained.

**Table 2:** Effect of neutralizing antibodies against angiogenic agents on HPMC-dependent proliferation of endothelial cells

	CM from young HPMCs		CM from senescent HPMCs	
	0.5 µM	10 µM	0.5 µM	10 µM
HUVEC				
3,3′,4,4′-THS + anti-VEGF	n.m.	62 ± 2*	n.m.	54 ± 9*
3,3′,4,4′-THS + anti-GRO-1/CXCL1	n.m.	102 ± 11	n.m.	98 ± 5
3,3′,4,4′-THS + anti-IL-8/CXCL8	n.m.	82 ± 6*	n.m.	81 ± 5*
3,3′,4,4′-THS + anti-MCP-1/CCL2	n.m.	94 ± 8	n.m.	103 ± 5
3,3′,4,4′,5,5′-HHS + anti-VEGF	84 ± 2*	58 ± 4*	76 ± 4*	65 ± 5*
3,3′,4,4′,5,5′-HHS + anti-GRO-1/CXCL1	92 ± 8	103 ± 5	89 ± 11	93 ± 6
3,3′,4,4′,5,5′-HHS + anti-IL-8/CXCL8	89 ± 6	72 ± 7*	89 ± 12	87 ± 3*
3,3′,4,4′,5,5′-HHS + anti-MCP-1/CCL2	94 ± 6	89 ± 6	92 ± 5	90 ± 10
HMVEC				
3,3′,4,4′-THS + anti-VEGF	n.m.	n.m.	n.m.	44 ± 6*
3,3′,4,4′-THS + anti-GRO-1/CXCL1	n.m.	n.m.	n.m.	89 ± 11
3,3′,4,4′-THS + anti-IL-8/CXCL8	n.m.	n.m.	n.m.	89 ± 5
3,3′,4,4′-THS + anti-MCP-1/CCL2	n.m.	n.m.	n.m.	99 ± 4
3,3′,4,4′,5,5′-HHS + anti-VEGF	86 ± 3*	72 ± 6*	56 ± 2*	67 ± 3*
3,3′,4,4′,5,5′-HHS + anti-GRO-1/CXCL1	95 ± 6	89 ± 12	89 ± 6	95 ± 2
3,3′,4,4′,5,5′-HHS + anti-IL-8/CXCL8	94 ± 3	93 ± 4	92 ± 7	99 ± 1
3,3′,4,4′,5,5′-HHS + anti-MCP-1/CCL2	102 ± 6	92 ± 6	91 ± 10	92 ± 5
HMEC-1				
3,3′,4,4′-THS + anti-VEGF	n.m.	56 ± 4*	71 ± 5*	56 ± 2*
3,3′,4,4′-THS + anti-GRO-1/CXCL1	n.m.	97 ± 4	92 ± 7	103 ± 2
3,3′,4,4′-THS + anti-IL-8/CXCL8	n.m.	43 ± 7*	54 ± 2*	48 ± 2*
3,3′,4,4′-THS + anti-MCP-1/CCL2	n.m.	97 ± 3	94 ± 5	99 ± 4
3,3′,4,4′,5,5′-HHS + anti-VEGF	n.m.	62 ± 8*	66 ± 6*	71 ± 4*
3,3′,4,4′,5,5′-HHS + anti-GRO-1/CXCL1	n.m.	89 ± 8	89 ± 7	101 ± 2
3,3′,4,4′,5,5′-HHS + anti-IL-8/CXCL8	n.m.	56 ± 3*	68 ± 3*	57 ± 7*
3,3′,4,4′,5,5′-HHS + anti-MCP-1/CCL2	n.m.	92 ± 5	92 ± 8	93 ± 2

The values are expressed as % of endothelial cell proliferation upon treatment with CM from HPMCs exposed to the stilbenes (considered as 100 %). In the case, where CM did not affect endothelial cell proliferation (see Fig. 2), studies with neutralizing antibodies were not performed (n.m.—not measured). The results derive from 8 experiments performed for each type of endothelial cells in duplicates. The asterisks indicate a significant decrease in endothelial cell proliferation

The results of studies on endothelial cell migration (presented in the Table 3) revealed that a significant inhibition of this process in HUVEC cultures was reached upon neutralization of IL-8/CXCL8 in CM derived from HPMCs exposed to both RVT analogues. In HMVECs exposed to CM from HPMCs treated with 3,3′,4,4′-THS this effect was observed in response to anti-IL-8/CXCL8 antibodies. In HMVECs exposed to CM from HPMCs treated with 3,3′,4,4′,5,5′-HHS as well as in HMEC-1 cultures, cell migration was effectively inhibited by neutralization of either IL-8/CXCL8 or VEGF. Similarly to results of cell proliferation, endothelial cell migration remained unchanged in cells exposed to the antibodies directed against GRO-1/CXCL1 and MCP-1/CCL2.

**Table 3:** Effect of neutralizing antibodies against angiogenic agents on HPMC-dependent migration endothelial cells

	CM from young HPMCs		CM from senescent HPMCs	
	0.5 µM	10 µM	0.5 µM	10 µM
HUVEC				
3,3′,4,4′-THS + anti-VEGF	n.m.	n.m.	n.m.	93 ± 9
3,3′,4,4′-THS + anti-GRO-1/CXCL1	n.m.	n.m.	n.m.	106 ± 15
3,3′,4,4′-THS + anti-IL-8/CXCL8	n.m.	n.m.	n.m.	46 ± 7*
3,3′,4,4′-THS + anti-MCP-1/CCL2	n.m.	n.m.	n.m.	101 ± 6
3,3′,4,4′,5,5′-HHS + anti-VEGF	n.m.	n.m.	n.m.	85 ± 8
3,3′,4,4′,5,5′-HHS + anti-GRO-1/CXCL1	n.m.	n.m.	n.m.	83 ± 14
3,3′,4,4′,5,5′-HHS + anti-IL-8/CXCL8	n.m.	n.m.	n.m.	65 ± 6*
3,3′,4,4′,5,5′-HHS + anti-MCP-1/CCL2	n.m.	n.m.	n.m.	92 ± 7
HMVEC				
3,3′,4,4′-THS + anti-VEGF	n.m.	n.m.	92 ± 5	88 ± 16
3,3′,4,4′-THS + anti-GRO-1/CXCL1	n.m.	n.m.	87 ± 13	88 ± 9
3,3′,4,4′-THS + anti-IL-8/CXCL8	n.m.	n.m.	73 ± 8*	56 ± 8*
3,3′,4,4′-THS + anti-MCP-1/CCL2	n.m.	n.m.	92 ± 4	93 ± 6
3,3′,4,4′,5,5′-HHS + anti-VEGF	n.m.	n.m.	n.m.	87 ± 3*
3,3′,4,4′,5,5′-HHS + anti-GRO-1/CXCL1	n.m.	n.m.	n.m.	91 ± 6
3,3′,4,4′,5,5′-HHS + anti-IL-8/CXCL8	n.m.	n.m.	n.m.	74 ± 6*
3,3′,4,4′,5,5′-HHS + anti-MCP-1/CCL2	n.m.	n.m.	n.m.	91 ± 6
HMEC-1				
3,3′,4,4′-THS + anti-VEGF	n.m.	47 ± 3*	98 ± 3	51 ± 9*
3,3′,4,4′-THS + anti-GRO-1/CXCL1	n.m.	97 ± 3	95 ± 4	99 ± 7
3,3′,4,4′-THS + anti-IL-8/CXCL8	n.m.	36 ± 3*	24 ± 5*	35 ± 4*
3,3′,4,4′-THS + anti-MCP-1/CCL2	n.m.	95 ± 6	96 ± 7	93 ± 5
3,3′,4,4′,5,5′-HHS + anti-VEGF	n.m.	81 ± 13	95 ± 6	65 ± 1*
3,3′,4,4′,5,5′-HHS + anti-GRO-1/CXCL1	n.m.	95 ± 5	93 ± 4	95 ± 6
3,3′,4,4′,5,5′-HHS + anti-IL-8/CXCL8	n.m.	31 ± 3*	35 ± 4*	38 ± 7*
3,3′,4,4′,5,5′-HHS + anti-MCP-1/CCL2	n.m.	96 ± 3	92 ± 6	93 ± 5

The values are expressed as % of endothelial cell migration upon treatment with CM from HPMCs exposed to the stilbenes (considered as 100 %). In the case, where CM did not affect endothelial cell migration (see Fig. 3), studies with neutralizing antibodies were not performed (n.m.—not measured). The results derive from 8 experiments performed for each type of endothelial cells in duplicates. The asterisks indicate a significant decrease in endothelial cell migration

### Effect of stilbenes on production of reactive oxygen species (ROS) by HPMCs

**Fig. 7:**
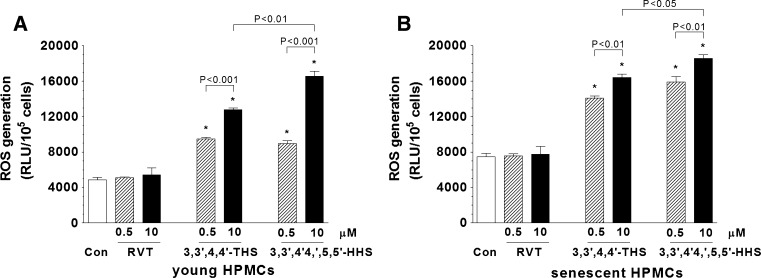
The effect of resveratrol (RVT), 3,3′,4,4′-THS and 3,3′,4,4′,5,5′-HHS on a generation of ROS by young (**A**) and senescent (**B**) HPMCs. The asterisks indicate a significant difference compared to the control group. The experiments were performed in duplicates with HPMC cultures derived from 6 different donors

The structure of 3,3′,4,4′-THS and 3,3′,4,4′,5,5′-HHS is characterized by the presence of the additional hydroxyl groups in the position *ortho*. Because such a position has been implicated in generating mitochondrial ROS, we designed the experiments in which young and senescent HPMCs were exposed to RVT (not possessing the *ortho* hydroxyl groups) and its derivatives for 4 h in order to compare the magnitude of ROS release upon exposure to these stilbenes. As shown in the Fig. 7, the release of ROS by HPMCs exposed to RVT (at the both concentrations) was comparable with control group. However, in cells exposed to 3,3′,4,4′-THS and 3,3′,4,4′,5,5′-HHS, the magnitude of ROS release was significantly enhanced. In the cells exposed to 10 μM of RVT analogues, the level of ROS generated in response to 3,3′,4,4′,5,5′-HHS was remarkably higher compared with 3,3′,4,4′-THS. The observed effects of the stilbenes on ROS release were similar in young and senescent HPMCs.

## Discussion

Angiogenesis, considered as a production of new blood vessels and/or improved permeability of the existing ones, plays an important role in various physiological and pathological processes, including embryonic vascular development, tissue repair/remodeling, diabetes, inflammation, and cancer. The central element in the development of blood vessels are endothelial cells which under control of strictly defined chemical mediators elicit degradation of vascular basement membrane, proliferate and migrate into the perivascular stroma which eventually leads to formation of tubular structures and tissue neovascularisation [27, 28].

In this paper we focused on the effects exerted by the natural (RVT) and synthetic (3,3′,4,4′-THS, 3,3′,4,4′,5,5′-HHS) stilbenes on the angiogenic activity of endothelial cells in vitro. In particular we examined proliferation and migration of endothelial cells in response to soluble factors released to an environment (CM) by young and senescent HPMCs. Such a model of angiogenesis studies, based on a co-culture system (employing endothelial cells and e.g. cancer cell-derived media [29, 30]), although rarer than a direct assessment of endothelial cell behaviour (addressed in this paper too), is of special value since it provides unique informations about the angiogenic activity controlled by the reciprocal interactions between different but nearby types of cells.

Endothelial cells are known to display a significant functional heterogeneity, also with respect to their angiogenic potential [31, 32]. Therefore in order to validate the research we used the cells derived from different vascular beds, namely umbilical vein-derived cells (HUVEC) representing macrovasculature, and microvascular endothelial cells originating from human dermis (HMVEC). Moreover, we employed an immortal dermal microvascular cells (HMEC-1) which are also commonly used in angiogenesis research [32], and which—as shown by Marchetti et al. [29]—are sensitive to growth-modulatory signals derived from non-autologous CM.

We were able to show that RVT and its synthetic derivatives exert contrary effects towards HPMC-dependent proliferation and migration of endothelial cells in vitro. Moreover we identified VEGF and the chemokine IL-8/CXCL8 as the prime agents whose production by HPMCs is differently modulated by stilbenes, and which are responsible for altered angiogenic responses of endothelial cells. We also evidenced that the changes in endothelial cell proliferation and migration, especially upon cell exposure to RVT analogues, are related to the replicative age of HPMCs. Last but not least we found that endothelial cell response to the stilbenes may vary depending on the type of endothelial cells employed.

First of all we observed that HPMCs treated with RVT create angiogenesis suppressive milieu which is reflected by inhibited proliferation and migration of endothelial cells in response to CM from either young or senescent HPMCs. To the best of our knowledge these findings are the first to show that RVT can modulate secretory phenotype in a certain type of normal somatic cells leading to decreased angiogenic activity of endothelial cells. Decreased production of VEGF and IL-8/CXCL8 upon treatment with RVT has already been described in various cell types, including fibroblasts [33], smooth muscle cells [34], epithelial cells [35], and melanoma-endothelial cell co-culture [36]. However, despite the convergence of these data with our own observations, a declined release of angiogenic factors by these cells has never been correlated with a concomitant anti-angiogenic response of endothelial cells. Interestingly, primary endothelial cells differed from the immortal ones with respect to the effective RVT concentration confirming differential responsiveness of these cells to angiogenesis-modulatory stimuli [32].

To mechanistically explain our observations we employed exogenous recombinant forms of angiogenic factors constitutively released by HPMCs and found that an attenuated proliferation and migration of endothelial cells in response to HPMC-derived CM is due to a down-regulated secretion of two potent angiogenic mediators, VEGF and IL-8/CXCL8, by HPMCs. Such a conclusion, especially pointing on a prominent role of VEGF, is in line with the results obtained by Srivastava et al. [14], who found that the neutralization of VEGF with specific antibodies may enhance the anti-angiogenic effects of RVT in HUVECs. These findings also agree with the results of an elegant study of Trapp et al. [36] who found, using melanoma-endothelial cell three-dimensional co-culture, that the anti-angiogenic activity of RVT may be caused by decreased release of VEGF by tumor cells.

To confront our results regarding an indirect (HPMC-dependent) effect of RVT on the endothelial cell angiogenic activity we also examined the direct impact of this compound on endothelial cell proliferation and migration. Our studies convincingly showed that RVT has an ability to inhibit both aspects of endothelial cell angiogenic behaviour, irrespectively on the type of endothelium used. This observation is in keeping with the results of previous studies on HUVECs and HMEC-1 cells [37–39], and may be attributed to an inhibition of MEK/ERK signaling pathway and concomitant activation of the FOXO transcription factor [14]. The anti-angiogenic activity of RVT in HUVECs may also be associated, as suggested by Wang et al. [40], with the activation of the glycogen-synthase kinase 3β (GSK3β) which results in down-regulation of β-catenin signaling pathway that eventually leads to decreased production of VEGF.

In contrast to a well-defined activity of RVT, the biological effects of its synthetic derivatives, also regarding their angiogenic activity, are still elusive. On the other hand, even the slight modifications of RVT structure, e.g. methylation of the hydroxyl groups or reduction of the double bond in the stilbenic skeleton have been found to efficiently change the functional properties of these new compounds, including an abolition of their antioxidative and/or antiangiogenic activity [41]. In this project we used two synthetic, additionally hydroxylated RVT analogues, 3,3′,4,4′-THS and 3,3′,4,4′,5,5′-HHS, which differ from their natural precursor in two features: the number of the hydroxyl (–OH) groups and the presence of the hydroxyl groups in a position *ortho*. Importantly, of these two derivatives, only 3,3′,4,4′,5,5′-HHS has been used so far in bio-medical research, and was appreciated as a strong anti-proliferative, cytotoxic, and pro-apoptotic agent with respect to plethora of malignant cells [18, 42, 43].

Our studies showed that the CM from HPMCs exposed to either 3,3′,4,4′-THS or 3,3′,4,4′,5,5′-HHS facilitated both proliferation and migration of all types of endothelial cells. Significantly enough, these effects were evident for endothelial cells exposed to CM from both young and senescent HPMCs, however, in the case of HMVEC i HMEC-1 cell proliferation as well as HUVEC and HMVEC migration, the observed angiogenic reactions were much more pronounced in response to CM from senescent HPMC cultures. This situation may result from an increased production of angiogenic agents by senescent HPMCs which phenomenon was described recently [8] as well as it was shown in this report again.

The measurements of the angiogenic agent concentration in HPMC-derived CM combined with the experiments with specific neutralizing antibodies against these factors revealed that the pro-angiogenic activity of 3,3′,4,4′-THS and 3,3′,4,4′,5,5′-HHS may be associated with increased production of VEGF and IL-8/CXCL8 by HPMCs, and is not related to the production of the other angiogenesis mediators, such as GRO-1/CXCL1 and MCP-1/CCL2. This in turn may imply that from the mechanistic point of view an altered behaviour of endothelial cells, especially the VEGF-dependent reactions, may be associated with phosphorylation of VEGF receptor-1 (VEGFR-1) and VEGF receptor-2 (VEGFR-2), and subsequent p38-dependent reorganization of the actin fibers [44–46]. The causative role of increased concentration of angiogenic mediators in HPMC-dependent increase in the endothelial cell angiogenic responses is in keeping with previous studies on HUVECs and HMEC-1 cells in which cell exposure to exogenous VEGF markedly improved their proliferation [29, 32] and the neutralization of IL-8/CXCL8 inhibited endothelial cell migration in response to cancer cell-derived CM [30].

A comparison of the effects exerted by the both RVT analogues showed that their magnitude was similar but not entirely the same. For example, 3,3′,4,4′,5,5′-HHS was able to affect HPMC-dependent HUVEC and HMVEC proliferation at 0.5 μM, which concentration in the case of 3,3′,4,4′-THS was not effective. This slight difference may suggest that the pro-angiogenic HPMC-dependent activity of these compounds is, at least to some extent, related to the number of the hydroxyl groups in the aromatic rings. At the same time, one may not exclude that the differences in the angiogenic factor release and the concomitant changes in HPMC-dependent endothelial cell proliferation and migration observed between RVT and its derivatives may be linked with the presence of the *ortho* hydroxyl groups in the structure of the latter. The experiments using a microsomal model showed these *ortho* compounds are able, in contrast to RVT, to form the cytotoxic semiquinones and thus stimulate additional oxygen consumption. This is possibly performed via redox cycling at expense of reducing equivalents transferred by cytochrome b5 and leads to the formation of ROS [18]. Moreover it has been shown that 3,3′,4,4′,5,5′-HHS may enhance cellular oxidative stress by decreasing activity of the major antioxidative enzymes: superoxide dismutase and catalase [47]. Our studies agree with the above-mentioned scenario since we were able to show that ROS production in young and senescent HPMCs exposed to the *ortho* RVT derivatives was remarkably higher compared to RVT itself. Because ROS are known to be partly responsible for VEGF and IL-8/CXCL8 production [48, 49], which relation has recently been shown also in HPMCs [50], it may therefore be hypothesized that the *ortho* RVT analogues can stimulate increased release of angiogenic agents and the endothelial cell angiogenic responses through oxidative stress-dependent mechanism.

The direct exposure of endothelial cells to 3,3′,4,4′-THS and 3,3′,4,4′,5,5′-HHS provided conflicting results, especially with respect to the behaviour of the particular cell types. Namely, in the case of HUVECs both RVT analogues induced cell proliferation (but not migration) while they failed to affect the response of endothelial cells of dermal origin. This effect may suggest that the angiogenic reactions to RVT analogues may depend, at least partly, on the vascular bed from which the endothelial cells derive. Indeed, the comparative studies on HUVECs and HMEC-1 cells showed that they may markedly differ with respect to various features associated with their angiogenic potential, including the basal proliferative capacity, magnitude of the angiogenic factor release, secretion of VEGFR-1, and response to exogenous growth factors [32].

The results we provided in this paper may have some clinical implications. In particular, decreased HPMC-dependent angiogenic reactions in response to RVT could be used in a prevention and/or a therapy of peritoneal cancer metastases [51]. Moreover they might also be employed in the context of peritoneal dialysis to prevent augmented peritoneum vascularization in patients continuously exposed to peritoneal dialysis fluids (PDFs). In fact, as shown in these patients, the long-term efficacy of peritoneal dialysis markedly declines—often leading to patients death—which is primarily attributed to a gradual loss of osmotic gradient and ultrafiltration failure, due to increased absorption of glucose (osmotic agent) through hypervascularised peritoneal membrane [52]. It has been evidenced that augmented vascularization of the peritoneum may be, in turn, caused by increased production of VEGF by HPMCs exposed to PDFs [53]. Since RVT is also appreciated due to its antioxidative and anti-inflammatory properties [54], its presence as an additive to dialysis solutions might have other beneficiary effects in reduction of high glucose-induced *peritonitis* [55], oxidative stress [56], tissue fibrosis [57], and premature mesothelial cell senescence [58]. On the other hand, our results pointing on the pro-angiogenic effects of RVT analogues should be considered with caution by those who working on these compounds (e.g. 3,3′,4,4′,5,5′-HHS) uncritically suggest their usefulness in the anti-cancer therapy [42, 43, 59]. The threat associated with the use of these stilbenes is related to stimulation of angiogenesis, especially in tissues which are known to accumulate senescent cells. These include, among others the omentum, in which the presence of senescent HPMCs was evidenced quite recently [5].

Taken together, our results indicate that RVT and its synthetic analogues may alter secretory phenotype of HPMCs in an opposite directions. These changes, leading to decreased (RVT) or increased (RVT analogues) efficacy of HPMC-dependent proliferation and migration of endothelial cells, should be considered in experimental and clinical approaches in which these stilbenes will be employed in prevention and/or therapy of certain pathologies. Further structure–activity studies are needed to determine the exact reasons of the disparate effects of natural and synthetic stilbenes on the biological properties of HPMCs. The molecular reasons of the different responsiveness of certain endothelial cell types to the stilbenes need also to be precisely delineated.
